# The Visual Analogue WOMAC 3.0 scale - internal validity and responsiveness of the VAS version

**DOI:** 10.1186/1471-2474-11-80

**Published:** 2010-04-30

**Authors:** Paula Kersten, Peter J White, Alan Tennant

**Affiliations:** 1 School of Health Sciences, University of Southampton, Highfield, Southampton, UK; 2 Department of Rehabilitation Medicine, Faculty of Medicine and Health, University of Leeds, Leeds, UK

## Abstract

**Background:**

Many people suffer with Osteoarthritis (OA) and subsequent morbidity. Therefore, measuring outcome associated with OA is important. The Western Ontario and McMaster Universities Osteoarthritis Index (WOMAC) has been a widely used patient reported outcome in OA. However, there is relatively little evidence to support the use of the Visual Analogue Scale (VAS) version of the scale. We aimed to explore the internal validity and responsiveness of this VAS version of the WOMAC.

**Methods:**

Patients with chronic hip or knee pain of mechanical origin, waiting for a hip or knee joint replacement completed the WOMAC as part of a study to investigate the effects of acupuncture and placebo controls. Validity was tested using factor analysis and Rasch analysis, and responsiveness using standardised response means.

**Results:**

Two hundred and twenty one patients (mean age 66.8, SD 8.29, 58% female) were recruited. Factor and Rasch analysis confirmed unidimensional Pain and Physical Functioning scales, capable of transformation to interval scaling and invariant over time. Some Differential Item Functioning (DIF) was observed, but this cancelled out at the test level. The Stiffness scale fitted the Rasch model but adjustments for DIF could not be made due to the shortness of the scale. Using the interval transformed data, Standardised Response Means were smaller than when using the raw, ordinal data.

**Conclusions:**

The WOMAC Pain and Physical Functioning subscales satisfied unidimensionality and ordinal scaling tests, and the ability to transform to an interval scale. Some Differential Item Functioning was observed, but this cancelled out at the test level and, by doing so, at the same time removed the disturbance of unidimensionality. The scaling characteristics of sets of items which use VAS require further analysis, as it would appear that they can lead to spurious levels of responsiveness and scale compression because they exaggerate the distortion of the ordinal scale.

**Trial number:**

UKCRN study ID: 4881

ISRCTN78434638

## Background

The prevalence of Osteoarthritis (OA) has been reported to be as high as 8.5 million people in the UK [1] and many patients suffer a considerable amount of pain and functional limitation [2,3]. Therefore, the evaluation of patients' health status is important in supporting individual treatment decisions and assessing quality of care and treatment [4,5]. In recent years we have seen an ever increasing number of patient reported outcome measures (PROMs) to aid in this process, which are now routinely used to monitor health care provision in the UK [4]. One commonly used measure in osteoarthritis is the Western Ontario and McMaster Universities Osteoarthritis Index (WOMAC) [6]. The scale has three subscales (Table 1), pain (5 items), stiffness (2 items) and physical functioning (17 items). Numerous studies have reported on its reliability and validity [7-9]. There have also been several studies, which have raised issues about the factorial validity of the subscales [5,10-13]. Evidence from research using the Rasch measurement model [14] seems to be consistent in observing a lack of fit to the Rasch model, a need to reduce the item set to achieve fit, or lack of confirmation of the distinct subscale structure [15-19]. It is unusual in offering a Likert-style version and a Visual Analogue Scale (VAS) version. However, much of the validation work appears to have been undertaken on the Likert version of the WOMAC. One study compared the Likert and VAS versions, suggesting differential efficiency for subscales depending on which versions were used [20]. The study did not report on factorial validity. Consequently, there is little evidence to support the reliability and factorial validity of the VAS version of the scale. Yet VAS's are increasingly used within scales and as single items in clinical practice and research. The VAS tends to be analysed as an interval scale but there is no scientific evidence that this is a reasonable assumption. The little evidence that exists to support the psychometric properties of the VAS scale suggests that they are ordinal, that people do not tend to use the full range of the scale, and that the actual design of the VAS can be different when measuring the same construct and thus could benefit from standardisation [21,22]. Further, if people do not use the full range of the VAS this might have implications for its responsiveness.

**Table 1:** WOMAC subscales descriptive data (raw scores and Rasch transformed scores)

Raw (ordinal data): scores are divided by 2 so scores can range from 0-50	Mean	SD	Range	Wilcoxon Signed Ranks test		Standardised Response Mean
				Z	P-value	

Pre Pain score	27.23	8.45	5.30 to 47.40	-6.543	<0.001	0.55
			
Post Pain score	22.43	10.41	0 to 45.90			

Pre Physical Functioning score	27.99	8.99	2.53 to 46.12	-5.187	<0.001	0.49
			
Post Physical Functioning score	24.47	9.99	2.79 to 47.62			

Pre Stiffness score	30.66	10.6	1.50 to 49.75	-4.531	<0.001	0.43
			
Post Stiffness score	27.08	11.45	0.25 to 47.25			

						

**Rasch transformed (interval data): the unit is in logits**	**Mean**	**SD**	**Range**	**Paired t-test**		**Standardised Response Mean**

				Z	P-value	

Pre Pain score	0.069	0.177	-0.503 to 1.010	4.802	<0.001	0.35
			
Post Pain score	-0.051	0.348	-4.410 to 0.510			

Pre Physical Functioning score	0.043	0.168	-0.717 to 0.631	4.465	<0.001	0.37

Post Physical Functioning score	-0.01	0.181	-0.740 to 0.850			

Pre Stiffness score	0.121	0.305	-0.905 to 1.971	4.627	<0.001	0.34
			
Post Stiffness score	0.026	0.297	-1.446 to 0.939			

Thus, whilst the WOMAC is a popular measure to assess impairment and activity limitation in patients with osteoarthritis, we lack evidence on the internal construct (factorial) validity of the VAS version [23]. In addition, further evidence on the extent to which the WOMAC VAS version can detect change over time (responsiveness) [24] is required. This paper examines the key concepts of internal validity and responsiveness of the WOMAC v3.0 VAS scale with factor and Rasch analysis.

## Methods

WOMAC v3.0 data (VAS version) were collected as part of a prospective randomised controlled trial, which investigated the relative effects of acupuncture and different acupuncture placebo controls on osteoarthritis (OA) patients waiting for hip or knee replacement. OA was diagnosed by orthopaedic consultants both clinically and radiographically. Patients were included if they had chronic pain predominantly from a single joint (hip or knee) of mechanical origin, and scored a minimum of 30 on a 100 mm VAS scale for pain, and were not on active treatment (apart from their normal analgesia). Those with serious co-morbidity (such as cancer, rheumatoid arthritis, severe low back pain), pregnant, prolonged or current steroid use, or waiting for a joint revision were excluded. WOMAC data was collected at two time points, on entry into the study and at the end, six weeks later.

### Data analysis

Given that the WOMAC is an established outcome measure with three subscales we conducted all analyses on each of the subscales. To avoid spurious precision, where the thickness of a mark upon a VAS may exceed one millimetre, or the interpretation of the exact location may vary by a millimetre, WOMAC data were divided by 2, thus reducing the range of each item to 0-50. For the purpose of this paper we will refer to these raw data as 'ordinal data'. Internal reliability of each of the subscales was examined with a Cronbach alpha, deemed acceptable for group use if >0.7 [25]. Also, each subscale was subjected to factor analysis where Monte-Carlo Parallel analysis was employed to determine significant eigenvalues [26]. Parallel analysis looks at the values of the eigenvalues as determined in a Monte-Carlo simulated random data set with the same sample size and number of items. It determines if the eigenvalue observed in the data is truly significant, given the generated random data. Default values in some statistical packages such as eigenvalues greater than one do not take this into account, and can generate spurious factors. Factor analysis and Cronbach Alpha's were carried out using SPSS15 [27].

Data were fitted to the Rasch measurement model to determine if the individual subscales satisfied the expectation of the Rasch model [14,28]. The RUMM2020 software was used for this purpose [29]. The Rasch model is a mathematical algorithm that expresses the probabilistic expectations of item and person performances/estimates [30]. Specifically, the probability of a correct response or endorsement is a logistic function of the difference between the person and item parameter. Where data satisfy the expectations of the Rasch model, the summed subscale scores can be transformed into interval scale measurement [31] (for the purpose of this paper we will refer to these Rasch transformed data as 'interval data'). A number of tests are performed to determine if the data meet the assumptions of the Rasch model. A summary chi-square interaction statistic should be non-significant, showing no deviation from model expectation. Person and item fit residuals should be within the range of +/- 2.5 and mean person/item fit residuals should be close to zero (values of zero indicate perfect fit) [28]. Individual item chi-squares should be non-significant (Bonferroni adjusted).

Inconsistent use of response options (disordered thresholds), item bias across groups of respondents (Differential item functioning, DIF), multidimensionality, or local dependence may contribute to misfit:

• The thresholds between response categories (i.e. the transition point between adjacent categories), where the probabilities of a response is equally likely, should reflect an increase in the underlying trait (e.g. pain). In the case of the VAS every millimetre (mm) is a response category, resulting in 100 thresholds. However, since we divided scores by two, the number of thresholds was reduced to 50. Disordered thresholds can be observed and dealt with by grouping response categories.

• The scale should be invariant and not be influenced by bias (Differential Item Functioning or DIF). For example we wish to see that people from different groups, with equal amounts of the underlying trait under investigation (i.e. pain, physical functioning or stiffness), respond to items in the same manner. This requirement of invariance is indicated by a non-significant ANOVA of the residuals where the key group is the main factor. DIF can be uniform and present consistently across the trait (see below how to deal with this), or non-uniform where bias is not consistent across the trait. Items which display non-uniform DIF often need to be removed from the scale [32,33]. Invariance across key groups (age, gender, joint affected, previous experience of acupuncture, which practitioner they were allocated to, and treatment allocation) was examined using an analysis of variance of the residuals where the group is the main effect.

• Unidimensionality is a requirement for summating any set of items [34]. It is examined by creating two subsets of items that are identified by a principal component analysis of the item residuals; those loading negatively forming one set and those loading positively the second set [35]. T-tests on the two estimates derived from the subtests for each respondent are then performed to see if they differ statistically; if the 95% confidence interval of the proportion of significant tests includes 5%, unidimensionality is supported [35,36].

• The correlation matrix of item residuals is explored to ensure that examinee item responses depend only on their trait level (local independence, residual correlations <0.30) and not on their responses to other test items.

Where items display uniform DIF they are grouped together into a testlet [37]. Essentially this combines the responses of the offending items into a 'super item'. Thus, we see if the bias is cancelled out at the test level and if so this allows an unbiased estimate of the person estimate. Similarly, where local dependency is found to exist, the locally dependent items are added into a testlet to explore if this removes the dependency in the data [37].

The person separation index (PSI) is an indicator of how precisely subjects have been spread out along the measurement construct defined by the items (ranges from 0 to 1) [28]. Values ≥0.70 allow for group comparisons but for individual clinical use values should be ≥0.85. If the scale is found to fit, we explore how well the scale is targeted to the sample, using item-person threshold maps.

For polytomous data two different parameterisations of the Rasch model can be used. The Rating Scale version assumes that the distance between thresholds is equal across items [38]. The Unrestricted (Partial Credit) model does not make this assumption [39]. If results from these two models are significantly different (using a log-likelihood test) the Partial Credit model should be used as was the case with our data (Pain subscale χ^2 ^= 53.84, p < 0.001; Physical Functioning subscale χ^2 ^= 206.83, p < 0.001; Stiffness subscale χ^2 ^= 19.47, p < 0.001). Bonferroni corrections were applied throughout the analysis to allow for multiple testing [40].

Responsiveness was examined using both the observed, ordinal scores on the VAS, and those derived from the Rasch analysis (log transformed interval data). For the latter purpose we obtained log transformed data both on the pre- and post data). Standardised Response Means (SRM) were used to evaluate the subscales' responsiveness. SRMs are derived by dividing the mean change score by the pooled standard deviation [41]. This accounts for different levels of variance in the data at baseline and follow-up. Bootstrapped standard errors were generated within the STATA programme to provide confidence intervals to ascertain if the difference between SRM's were significantly different [42].

#### Ethics

Ethics approval was gained from the Southampton & South West Hampshire and the Salisbury and South Wiltshire Research ethics Committees (approval number 170/03/t).

## Results

221 Patients took part in the study (mean age 66.8, SD 8.3; 58% female, 42% male; 40% hip OA; 60% knee OA). Their median VAS pain score (over seven days before the commencement of the study) was 59.4 (IQR 48.0 to 68.9). Table 1 displays participants' raw scores (ordinal data) on each of the subscales, pre and post, and demonstrates that significant changes occurred over time on all subscales.

### Pain subscale

Factor analysis of the WOMAC Pain subscale (pre-data) demonstrated a unidimensional construct, with 70.6% of the variance attributable to the first factor.

Fit to the Rasch model was demonstrated by satisfactory summary statistics and t-tests for unidimensionality (table 2, analysis 1). Individual item fit was good. There were no significant residual correlations between the items suggesting absence of local dependence. Only two out of the five items were disordered (item 3 & 4). However, due to the large number of response categories (i.e. 51) it was not possible to determine a sensible rescoring method. The PSI of the pain subscale was 0.86 and Cronbach alpha was 0.82.

**Table 2:** Rasch analysis WOMAC subscales (pre data)

Analysis number	Item fit residual		Person fit residual		χ^2 ^interaction		PSI	Uni-dimensionality Independent t-test (95% CI)
**Pain subscale**	**Mean**	**SD**	**Mean**	**SD**	**Value (df)**	P*		

Pre-data								

Analysis 1	0.538	1.101	-0.433	1.224	5.65 (10)	0.844	0.86	6.3% (3.5 to 9.2)

Analysis 2	-0.065	2.811	-0.538	1.194	8.15 (8)	0.419	0.85	4.5% (1.7 to 7.4)

**Physical Functioning subscale**								

Analysis 3	0.478	1.511	-0.557	1.896	63.37 (34)	0.002	0.96	9.5% (6.6 to 12.4)

Analysis 4	0.757	1.393	-0.469	1.823	44.81 (32)	0.066	0.96	7.7% (4.8 to 10.6)

**Stiffness subscale**								

Analysis 5	-0.546	0.181	-0.499	0.722	8.61 (4)	0.072	0.81	2.3% (-0.6 to 5.2)

Pain subscale: Analysis 1: fit statistics of Pain subscale; Analysis 2: in this analysis we have combined biased items 2 and 4 into a testlet, the remaining items are unchanged.

Physical Functioning subscale: Analysis 3: fit statistics of Physical Functioning subscale; Analysis 4: in this analysis we have combined biased items 1 and 5 into a testlet, the remaining items are unchanged.

* Pain subscale Bonferroni adjusted (0.05/5 items, p-value considered significant if <0.01); Physical Functioning subscale Bonferroni adjusted (0.05/17 items, p-value considered significant if <0.003); Stiffness subscale Bonferroni adjusted (0.05/17 items, p-value considered significant if < 0.025)

Two items (2 and 4) showed uniform DIF by 'joint' in opposite directions: people with the same level of pain tended to score higher on item 2 if they were waiting for a knee replacement than those waiting for a hip replacement. The reverse was the case for item 4. Combining these two items into a testlet and comparing them against the remaining three items resulted in a fit to the Rasch model and unidimensionality (table 2, analysis 2). This is an indication that the DIF is cancelled out at the subtest level. The resulting item fit statistics are shown in table 3.

**Table 3:** WOMAC items fit statistics

	Location	Standard Error	Fit Residual	χ^2^	DF	Probability
**Pain subscale items**	-0.050	0.010	-4.110	6.990	2	0.030

Item 2 and 4 combined into a testlet 2. Going up or down stairs 4. Sitting or lying	0.010	0.010	1.430	0.240	2	0.885

1. Walking on a flat surface	0.050	0.010	0.260	0.100	2	0.953

3. At night while in bed	0.000	0.010	2.170	0.820	2	0.663

5. Standing upright	-0.050	0.010	-4.110	6.990	2	0.030

**Physical Functioning subscale items**						

Item 1 and 5 combined into a testlet 1. Descending the stairs 5. Bending to floor	-0.350	0.006	4.267	6.574	2	0.037

2. Ascending the stairs	-0.101	0.009	1.135	0.466	2	0.792

3. Rising from sitting	-0.019	0.009	0.031	0.224	2	0.894

4. Standing	0.015	0.009	2.333	3.379	2	0.185

6. Walking on flat	0.065	0.009	1.055	0.020	2	0.990

7. Getting in/out of car	-0.035	0.009	-0.875	6.211	2	0.045

8. Going shopping	-0.024	0.009	-0.379	2.174	2	0.337

9. Putting on socks/stockings	-0.060	0.008	2.018	1.134	2	0.567

10. Rising from bed	0.034	0.008	0.146	7.781	2	0.020

11. Taking off socks/stockings	-0.021	0.008	-0.658	0.652	2	0.722

12. Lying in bed	0.112	0.008	1.606	1.334	2	0.513

13. Getting in/out of bath	-0.058	0.008	1.475	1.433	2	0.488

14. Sitting	0.194	0.009	0.467	2.195	2	0.334

15. Getting on/off toilet	0.132	0.008	0.290	2.405	2	0.300

16. Heavy domestic duties	-0.096	0.009	-1.238	8.452	2	0.015

17. Light domestic duties	0.212	0.009	0.433	0.371	2	0.831

**Stiffness subscale items**						

1. How severe is your stiffness after first wakening in the morning?	-0.026	0.010	-0.674	4.113	2	0.128

2. How severe is your stiffness after sitting, lying or resting later in the day?	0.026	0.010	-0.418	4.495	2	0.106

* Pain subscale Bonferroni adjusted (0.05/5 items, p-value considered significant if <0.01); Physical Functioning subscale Bonferroni adjusted (0.05/17 items, p-value considered significant if <0.003); Stiffness subscale Bonferroni adjusted (0.05/17 items, p-value considered significant if < 0.025)

**Figure 1:**
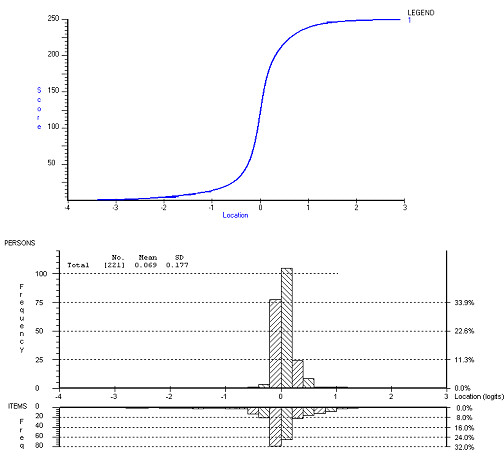
**Item Response Function for the WOMAC Pain subscale (pre-data) and Person-Item-Threshold map**. The Figure displays both the Item Response Function for the WOMAC Pain subscale and the Person-Item threshold map to illustrate the location of the item thresholds (clustered closely together along the bottom half of the lower part of the diagram) and the consequences for the response function of the scale (upper half of the diagram). The y-axes display the raw scores (top y-axis) which range from 0 to 250 as we divided the VAS scores by half for the analysis and the subscale contains five items, and the frequencies of item thresholds and participants (bottom y-axes). The Figure also shows the location of study participants along the construct of Pain. Data for this figure represent the unbiased person estimates derived from Analysis 2 (see also Table 1) which combined biased items 2 and 4 into a testlet and left the remaining items are unchanged.

Despite the potential 250 raw score points (ordinal data) derived from the 5 items, the scale demonstrated a substantial lack of range (figure 1). This is consistent with the moderate reliability and indicates that increments in raw (ordinal) score points across the centre of the scale are associated with only marginal increments on the underlying metric construct (interval data).

There was absence of DIF over time when the pre- and post data were combined indicating that the scale is invariant by time and the items were well targeted to the population. The SRM for the ordinal data (raw scores) was 0.55 and for the interval (Rasch transformed scores) data 0.35 suggesting the ordinal SRM is overestimating the true responsiveness of the WOMAC (table 1). However, the confidence interval for the difference between the two SRM's overlapped zero, indicating that the difference was not significant.

### Physical Functioning (PF) subscale

Factor analysis of the 17 item PF subscale supported a unidimensional construct, with 63.4% of the variance attributable to the first (and only significant) factor.

The pre-data PF items initially deviated significantly from the Rasch model with a chi-square probability >0.003 (table 2, analysis 3) and a lack of unidimensionality. Five items showed significant DIF by joint (item 1, 2, 5, 9 and 11). In addition, items 1 and 5 had high fit residuals. As these two items also showed DIF they were combined into a testlet and compared with the remaining 15 items. This resulted in a fit to the Rasch model and unidimensionality (table 2, analysis 4; table 3), suggesting DIF was responsible for the lack of fit and unidimensionality. Cronbach alpha was 0.95.

**Figure 2:**
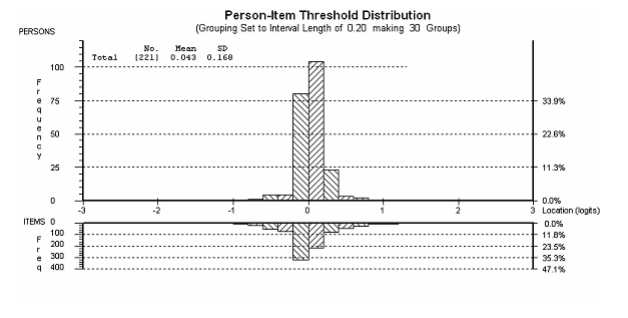
**Person-Item Threshold Response Map for the WOMAC Physical Functioning subscale (pre-data)**. The graph displays the person-item threshold distribution map with the x-axes displaying location or difficulty of item thresholds (lower half) and location or level of physical functioning reported by participants (upper half). The y-axes display the frequencies of item thresholds (lower half) and participants (upper half). Data for this figure represent the unbiased person estimates derived from Analysis 2 (see also Table 2) which combined biased items 1 and 5 into a testlet and left the remaining items are unchanged.

As with the pain scale, the PF scale (Rasch transformed scores) had a limited distribution (figure 2) and the ordinality of the raw score was accentuated. For example, a change in 25 points out of a total of 850 (17 items each ranging from 0-50 as scores were halved) at the margins of the raw total (ordinal) VAS physical functioning subscale scores is reflected in a real, interval equivalent change of 311 points (622 mm) (table 4). By contrast, a change of 25 ordinal points (50 mm) in the middle of the scale is in actual fact a change of only 2.2 interval points (4.4 mm).

**Table 4:** Total scores WOMAC Pain and Physical Functioning subscales: first 25 raw score (ordinal) points*

Raw total Pain VAS score (ordinal data)	Rasch transformed Pain VAS score (interval data)	Raw total Physical Functioning VAS score (ordinal data)	Rasch transformed Physical Functioning VAS score (interval data)
0	0.00	0	0
1	20.77	1	79.04
2	35.14	2	129.94
3	45.13	3	161.34
4	53.51	4	184.08
5	59.50	5	201.40
6	65.10	6	215.48
7	70.69	7	227.39
8	75.08	8	236.05
9	81.07	9	244.71
10	83.47	10	252.29
11	87.06	11	258.79
12	91.45	12	265.29
13	93.45	13	270.70
14	95.45	14	275.03
15	97.44	15	279.36
16	99.84	16	283.69
17	101.44	17	288.02
18	103.03	18	291.27
19	104.63	19	294.52
20	106.23	20	297.77
21	107.43	21	299.94
22	108.63	22	303.18
23	109.82	23	305.35
24	110.62	24	307.52
25	111.82	25	310.76

* Scores were halved for the analysis, therefore the pain total score ranges from 0-250 and the physical functioning total score from 0-850

The person fit residual standard deviation was high. We used a regression analysis to explore independent variables that might be predictive of this. Variables entered into this analysis were gender, age, joint and time on the waiting list. None correlated significantly with the person fit residuals.

Combining pre- and post data showed that the Physical Functioning Subscale was invariant over time (no DIF observed). The SRM using the ordinal data was 0.49 and using the interval data 0.37 (table 1). In this instance the confidence interval for the difference between SRM's did not overlap zero (0.017-0.206), indicating a significantly different effect size.

### Stiffness subscale

Since the stiffness subscale consists of two items it was not appropriate to subject it to Factor analysis. Rasch analysis showed that the subscale fitted the Rasch model (table 2, analysis 5). The reliability of this subscale was low (0.81), which is not unexpected considering the shortness of the scale. Cronbach alpha was 0.80. The subscale was invariant over time. Responsiveness using the interval data was again lower than that derived from the ordinal data (0.34 versus 0.43, table 1). On this occasion this difference was non-significant, with the confidence interval for the difference overlapping zero.

## Discussion

Data from the three WOMAC subscales were assessed by factor and Rasch analysis, which largely supported the structure of the Pain and Physical Function subscales. There was some bias in item response, but this tended to cancel out at the scale level. Of significance is that this bias, when uncorrected, gave rise to the appearance of multidimensionality, and misfit to the Rasch model. This is consistent with earlier findings about the impact of DIF on dimensionality [43]. It is thus possible that earlier Rasch analyses of the WOMAC, which did not adjust for this bias, may have indicated that item reduction was necessary to obtain fit to the model and/or unidimensionality [15-19]. The use of testlets as a mechanism to evaluate the potential cancelling effect of bias appears to be a useful strategy to avoid unnecessary and possibly incorrect item deletion.

Classical factor analysis may also have led to a conclusion of multidimensionality if parallel analysis was not applied [10-13]. In the current analysis, the default rule of an eigenvalue of greater than one as significant would have led to a multidimensional solution for the Physical Function scale. Although many items cross loaded across two factors, at least two items would have been candidates for removal under these circumstances. Therefore, it is easy to see how slight differences in methodological approaches may have given rise to different solutions regarding the subscale structures of the WOMAC.

In addition, the inclusion of OA patients in different stages of their disease in other studies may have given rise to valid multidimensional conclusions and consequently careful testing of the structure of scales across all stages (and disease groups) is a prerequisite for confidence in the robustness of any generic scale [44].

Although the stiffness subscale only consists of two items it was shown to fit the Rasch model. However, we were not able to employ strategies to overcome observed DIF and reliability was low. The usefulness of this scale should therefore be reconsidered.

The Rasch model is strict in terms of satisfying the requirement for transformation to interval scaling [45,46]. The iterative process of Rasch analysis requires unidimensionality tests to be done at each stage. Thus, factor analysis and Rasch analysis provide their own hierarchical ordering of scalability with the assumption of unidimensionality and finally the potential for interval scale transformation. The WOMAC Pain and Physical Function scales satisfy all of these conditions in this sample of those awaiting hip or knee replacement.

Responsiveness of the WOMAC has been reported to be good, both for the Likert and the VAS versions [47-52]. However, these studies make no attempt to adjust for the ordinal nature of the Likert scale or VAS, and the resulting differential deviation from the interval scale metric. As the calculation of responsiveness involves mathematical operations which are not supported by ordinal data, the results based upon ordinal data may be spurious [53]. Clinicians and others may be tempted to choose the VAS version of the scale because it seems more responsive than a Likert version. Figure 1 showed that a wide range of ordinal raw score points in the middle of the score range are associated with a very small number of actual metric points, and that at the margins the converse is true. In other words, the distance between data points in the middle of a visual analogue scale (in millimetres) as deduced from the raw (ordinal) data is in fact much smaller once data are transformed into interval level data and thus the calculation of the SRM provides a good example of the impact of the misuse of ordinal data. Consequently, the level of responsiveness is spurious, as evidenced by the fall in SRM on all subscales when calculated using the interval data (where the technique is valid). Therefore, when using raw ordinal data researchers and clinicians run the risk of misinference, regarding the magnitude of change in pain and physical functioning [54]. Other studies employing Rasch analyses of visual analogue scales have not reported on the logit range and we can therefore not compare these findings to others. Further work needs to be undertaken to evaluate the effect of scale units (i.e. ordinal versus interval) upon statistics such as the SRM, and upon routine interpretation of outcome.

There are a number of limitations to the study. The sample is taken from those awaiting arthroplasty and therefore may be reflective of only those with moderate or severe pain and functional limitations. Consequently the findings need replication in those with lesser severity. The high person fit residual SD found in the Physical Functioning subscale was puzzling and could not be explained by the effects of a number of independent variables such as gender, age, time on the waiting list and joint. It is possible that these may also be a function of the large number of data points, and the associated sample size and again this will require further work.

## Conclusions

In conclusion, the WOMAC Pain and Physical Functioning subscales were found to fit Rasch model expectations, and thus be internally valid and unidimensional. Factor analysis using parallel analysis also confirmed the unidimensionality. Consequently the raw score is a sufficient statistic for estimating the person's level of pain and physical functioning at the ordinal level. We were also able to transform the ordinal data (constrained to a 0-50 range for each item) to an interval scale through fit to the Rasch model. Some Differential Item Functioning was observed, but this cancelled out at the test level and, by doing so, at the same time removed the disturbance of unidimensionality. Therefore, we do not recommend changes to the item structure of the subscales. However, the scaling characteristics of sets of items which use Visual Analogue Scales do require further analysis, as it would appear that responsiveness using ordinal data is under-reported when people move along the margins of the scale and over-reported when they move across the middle of the scale. Clinically this means that change over time on the WOMAC for patients on the margins, using the raw ordinal data, cannot be directly compared with those who score in the middle of the scale, consistent with the lack of validity of performing mathematical operations on ordinal data. Finally, the utility of the Stiffness subscale should be reconsidered.

## Competing interests

The authors declare that they have no competing interests.

## Authors' contributions

PW conceived of the study, was responsible for its design and coordination and helped to draft the manuscript. PK performed the analyses and drafted the manuscript. AT supported the analysis and manuscript draft. All authors read and approved the final manuscript.

## Pre-publication history

The pre-publication history for this paper can be accessed here:

http://www.biomedcentral.com/1471-2474/11/80/prepub
